# Machine Learning for 5G MIMO Modulation Detection

**DOI:** 10.3390/s21051556

**Published:** 2021-02-24

**Authors:** Haithem Ben Chikha, Ahmad Almadhor, Waqas Khalid

**Affiliations:** 1 Computer Engineering and Networks Department, College of Computer and Information Sciences, Jouf University, Sakaka 72388, Saudi Arabia; aaalmadhor@ju.edu.sa; 2 Institute of Industrial Technology, Korea University, Sejong 30019, Korea; waqas283@korea.ac.kr

**Keywords:** 5G, multi-relay cooperative MIMO systems, modulation detection, random committee machine learning technique

## Abstract

Modulation detection techniques have received much attention in recent years due to their importance in the military and commercial applications, such as software-defined radio and cognitive radios. Most of the existing modulation detection algorithms address the detection dedicated to the non-cooperative systems only. In this work, we propose the detection of modulations in the multi-relay cooperative multiple-input multiple-output (MIMO) systems for 5G communications in the presence of spatially correlated channels and imperfect channel state information (CSI). At the destination node, we extract the higher-order statistics of the received signals as the discriminating features. After applying the principal component analysis technique, we carry out a comparative study between the random committee and the AdaBoost machine learning techniques (MLTs) at low signal-to-noise ratio. The efficiency metrics, including the true positive rate, false positive rate, precision, recall, F-Measure, and the time taken to build the model, are used for the performance comparison. The simulation results show that the use of the random committee MLT, compared to the AdaBoost MLT, provides gain in terms of both the modulation detection and complexity.

## 1. Introduction

Recently, the integration of 5G new radio (NR) and multiple-input multiple-output (MIMO) has received increasing attention due to its effectiveness in improving both the capacity and robustness of the wireless systems [1]. In fact, the use of multiple antenna elements in MIMO systems is considered as one of the most promising technologies in 5G NR systems that can be employed to enable beamforming and spatial multiplexing [2]. The cooperative MIMO systems also offer a considerable rate gain and improve the diversity order [3,4,5,6,7]. The efficient relaying mechanisms are taken into account by the standard specifications of the mobile broadband communication systems, such as LTE-advanced (LTE-A) [8]. Furthermore, the estimation of communication parameters (e.g., number of antennas, coding, and modulation) has received a great deal of attention. It has found applications in several military and civilian communication systems, such as software-defined radio and cognitive radios [9,10]. It is important to consider the effect of spatial correlation and imperfect channel state information (CSI) in the cooperative MIMO systems. The distance limitation between the antennas and scatterers existing in propagation environment affects the diversity, multiplexing, and capacity gains. The errors caused by the channel estimation, quantization, reciprocity mismatch, and delay produce the imperfect CSI [11]. It is practically unfeasible to obtain the perfect CSI at all nodes [12].

### 1.1. Related Works

In a MIMO destination node, the decoder or the spatial demultiplexer and the demodulator are employed to recover the transmitted binary information. In fact, the destination node is the entity that converts the received waves into a binary stream. Therefore, the estimation of the transmitted binary information requires a prior knowledge of the communication parameters including the number of the source antennas, the noise variance, the coding, the channel matrix, and the modulation. To design powerful cognitive radio, many estimation algorithms of the communication parameters have been developed in the literature. In [13,14,15], the authors have proposed algorithms for estimating the number of the source antennas. Furthermore, several approaches have been proposed for the detection of the coding [16,17] and other algorithms for estimating the channel matrix are also available [18,19,20]. In the last few decades, there has been a great interest on modulation detection for MIMO communications systems [21,22,23,24,25,26]. In fact, the misdetection of the modulation type causes a considerable performance degradation in the estimation of source information as the modulation estimation is done prior to the demodulation phase. The current work is focusing on the modulation detection for cooperative MIMO communication systems. In order to alleviate the aforementioned concerns, the use of artificial intelligence (AI) to detect modulations, based on MLTs, is employed in existing literature as it is simple to implement and achieves a quasi-optimal performance when choosing an appropriate features set. Based on a MLT, the authors of [24] have investigated the modulation detection for the MIMO relaying system under the conditions of perfect CSI and uncorrelated channels. In [23], Lau et al. proposed an algorithm dedicated for the short-range devices that use the European 868 MHz band under the same conditions, i.e., perfect CSI and uncorrelated channels. Under the same conditions, the detection of superposed modulations for two-way relaying MIMO systems with the physical-layer network coding is studied in [22]. While there have been considerable works that assumed the conditions of the perfect CSI and uncorrelated channels, little attention has been paid to the cooperative MIMO systems with imperfect CSI and under correlated channels. In [21], we proposed an algorithm for the modulation detection dedicated to cooperative MIMO systems over the imperfectly estimated correlated channels. In this work, we consider the AdaBoost MLT; it provides good performance of the modulation detection at an acceptable signal-to-noise ratio (SNR). However, it suffers from large training time and convergence speed that can affect the design of practical modulation detection algorithm dedicated to multi-relay cooperative MIMO systems.

### 1.2. Contributions

In this study, we aim to propose a detection modulation algorithm with a low complexity that is dedicated to multi-relay cooperative MIMO systems over the imperfectly estimated correlated channels. More specifically, the contributions of this work are summarized as follows.

- We proposed a modulation detection using random committee MLT for multi-relay cooperative MIMO systems over the imperfectly estimated correlated channels. To the best of the authors knowledge, this is the first time that random committee MLT is used for the modulation detection. The purpose is to detect the modulation types and orders among different M-ary shift-keying linear modulations (M-PSK and M-QAM) used by broadband technologies, especially 5G NR.
- The modulation detection algorithms proposed in [21,24] are considered as benchmarks, and the comparative study is provided. The performance of the proposed modulation detection algorithm is investigated and evaluated with the number of efficiency metrics, such as the true positive rate, false positive rate, precision, recall, F-Measure, and the time taken. The superiority of the proposed modulation detection algorithm in terms of computational complexity and modulation detection is verified through the simulation results.

### 1.3. Outline

The rest of the paper is organized as follows. Section 2 presents the system model, including the system description and assumptions. The proposed modulation detection algorithm is provided in Section 3. The simulation results, along with the discussion and the benefits of the proposed algorithm, are given in Section 4. Finally, the conclusions of the paper are presented in Section 5.

### 1.4. Notations

In this paper, we use $tr\left(\cdot \right)$, $\bar{\left(\cdot \right)}$, ${\left(\cdot \right)}^{H}$, ${\left(\cdot \right)}^{T}$, and ${\left(\cdot \right)}^{-1}$ to denote the trace, conjugate, conjugate transpose, transpose, and inverse, respectively. ${\left(.\right)}_{r,c}$ represents the entry in the $r^{th}$row and the $c^{th}$ column of a matrix. $E\left[.\right]$ denotes the statistical expectation. $I_{N}$ stands for a N×N identity matrix. The set of M×N matrices over complex field is denoted by $C^{M\times N}$. Finally, CN(m,Σ) is a circularly symmetric complex Gaussian distribution with mean *m* and covariance matrix Σ. It is noted that the abbreviations used in this paper are listed in Table 1.

## 2. System Model and Assumptions

### 2.1. System Model

**Figure 1:**
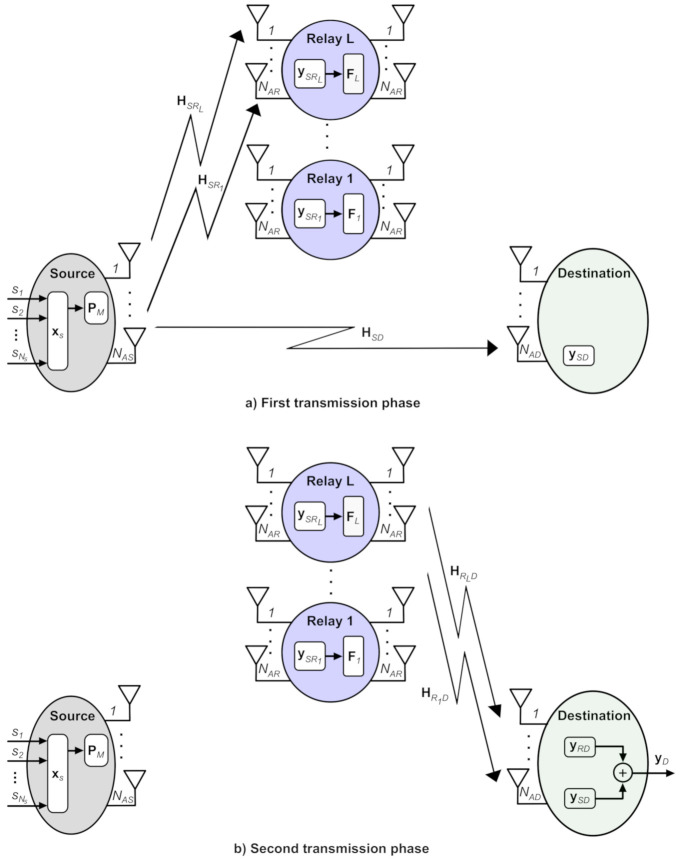
Multi-relay cooperative MIMO systems using one source, one destination and *L* relays.

We consider the multi-relay cooperative MIMO system over the spatially-correlated channel as shown in Figure 1. Here, we denote the source node by *S*, the destination node by *D*, and *L* relay nodes by $R_{l}$, l=1,2,…,L to make information transmission from *S* to *D*. We suppose that antennas $N_{AS}$, $N_{AR}$, and $N_{AD}$ are enabled at *S*, each $R_{l}$, and *D*, respectively. We apply a two-phase transmission protocol to allow the information transmission from *S* to *D*; via the direct link SD and the cooperative links ${SR}_{l}-R_{l}D,l=1,\ldots ,L$. To this end, a non-regenerative and half-duplex relay technique is employed for processing and forwarding the received signals at each $R_{l}$ [27]. Now, if we apply a spatial multiplexing (SM) at *S* and if all $R_{l}$ and *D* are to simultaneously support all the $N_{AS}$ independent substreams, then we must meet the requirements

(1) $$\left\{\begin{array}{c} N_{AD}\geq N_{AS},\\ N_{AR}\geq N_{AS}. \end{array}\right.$$

We presume that all nodes have an equal number of antennas for simplicity, i.e., $N_{AS}=N_{AR}=N_{AD}=N_{A}$.

We encode the source signals $x_{s}$ in the first transmission phase using SM. Therefore, the signals $x_{s}$ is given by

(2) $$x_{s}={\left[s_{1},s_{2},\ldots ,s_{N_{A}}\right]}^{T},$$

where $s_{1},s_{2},\ldots ,s_{N_{A}}$ are assumed to be independent, identically distributed (i.i.d.), and mutually independent. Given the fact that we stand the transmit power by $P_{x_{s}}$ at *S*, $x_{s}$ should satisfy the power constraint given by

(3) $$E\left[x_{s}x_{s}^{H}\right]=P_{x_{s}}{\left(N_{A}\right)}^{-1}I_{N_{A}}.$$

To achieve a near-capacity at sum-rate [28], we apply a regularized zero forcing (RZF) linear precoding technique at *S*. Consequently, the linear precoding matrix can be expressed as

(4) $$P_{M}={\hat{H}}_{SD}^{H}{\left({\hat{H}}_{SD}{\hat{H}}_{SD}^{H}+\alpha_{1}I_{N_{A}}\right)}^{-1},$$

where ${\hat{H}}_{SD}\in C^{N_{A}\times N_{A}}$ is the matrix that estimate the transmission channel from *S* to *D* with a Gaussian distributed error, and $\alpha_{1}$ represents the total noise variance to the total transmit power ratio expressed by [28]

(5) $$\alpha_{1}=N_{A}\sigma_{SD}^{2}/P_{x_{s}}.$$

After performing the RZF to $x_{s}$, *S* can transmit the precoded data in parallel to *D* and all $R_{l}$. At *D*, the received signal can be expressed as

(6) $$\begin{array}{ccc} y_{SD} & = & \rho_{s}H_{SD}P_{M}x_{s}+n_{SD}, \end{array}$$

where $\rho_{s}$ represents the control factor of the source power, given by

(7) $$\rho_{s}=\sqrt{P_{x_{s}}/\mathrm{tr}\left(P_{M}P_{M}^{H}\right)},$$

$H_{SD}\in C^{N_{A}\times N_{A}}$ is the SD channel matrix with spatial correlation and $n_{SD}∼\mathrm{CN}\left(0,\;\sigma_{SD}^{2}I_{N_{A}}\right)$ represents an additive zero-mean spatially-white circularly complex Gaussian noise with variance $\sigma_{SD}^{2}$. At $R_{l},l=1,\ldots ,L$, the received signal is given by

(8) $$\begin{array}{ccc} y_{{SR}_{l}} & = & \rho_{s}H_{{SR}_{l}}P_{M}x_{s}+n_{{SR}_{l}}, \end{array}$$

where $H_{{SR}_{l}}\in C^{N_{A}\times N_{A}}$ is the ${SR}_{l}$ channel matrix with spatial correlation and $n_{{SR}_{l}}∼\mathrm{CN}\left(0,\;\sigma_{{SR}_{l}}^{2}I_{N_{A}}\right)$.

In the second transmission phase, all $R_{l}$ apply a linear beamforming matrix (BM) to received signals from the first transmission phase. We model the linear BM according to the zero forcing and regularized zero forcing (ZF-RZF) [29], denoted by $F_{l}$ and given by

(9) $$\begin{array}{ccc} F_{l} & = & {\hat{H}}_{R_{l}D}^{H}{\left({\hat{H}}_{R_{l}D}{\hat{H}}_{R_{l}D}^{H}+\alpha_{2}I_{N_{A}}\right)}^{-1}\times \\ \; & \; & {\left({\left(H_{{SR}_{l}}P_{M}\right)}^{H}H_{{SR}_{l}}P_{M}\right)}^{-1}{\left(H_{{SR}_{l}}P_{M}\right)}^{H}, \end{array}$$

where ${\hat{H}}_{R_{l}D}\in C^{N_{A}\times N_{A}}$ is the matrix that estimate the transmission channel from $R_{l}$ to *D* with a Gaussian distributed error at *l*th relay node, and $\alpha_{2}$ represents the total noise variance to the total transmit power ratio expressed by [28]

(10) $$\alpha_{2}=N_{A}\sigma_{RD}^{2}/P_{r}.$$

After that, the resulting signals after ZF-RZF precoding are forwarded from all $R_{l}$, and the received signal at *D* can be written as

(11) $$\begin{array}{ccc} y_{RD} & = & \overset{L}{\sum_{l=1}}\rho_{r_{l}}\rho_{s}H_{R_{l}D}F_{l}H_{{SR}_{l}}P_{M}x_{s}+\overset{L}{\sum_{l=1}}\rho_{r_{l}}H_{R_{l}D}F_{l}n_{{SR}_{l}}+n_{RD}, \end{array}$$

where $\rho_{r_{l}}$ represents the control factor of the *l*th relay power, given by

(12) $$\rho_{r_{l}}=\sqrt{P_{r}/\mathrm{tr}\left(\rho_{s}^{2}F_{l}H_{{SR}_{l}}P_{M}P_{M}^{H}H_{{SR}_{l}}^{H}F_{l}^{H}+\sigma_{RD}^{2}F_{l}F_{l}^{H},\right)}$$

and $H_{R_{l}D}\in C^{N_{A}\times N_{A}}$ is the $R_{l}D$ channel matrix with spatial correlation and $n_{R_{l}D}∼\mathrm{CN}\left(0,\;\sigma_{R_{l}D}^{2}I_{N_{A}}\right)$.

### 2.2. Spatial Correlation Model

The distance limitation between the antennas and scatterers existing in the propagation environment can affect the diversity, multiplexing, and capacity gains [30]. We propose to model spatial correlation for cooperative MIMO channels based on the Kronecker model [30]. Accordingly, the channel correlation matrices $H_{SD}$, $H_{{SR}_{l}}$, and $H_{R_{l}D}$ can be expressed as

(13) $$\left\{\begin{array}{c} H_{SD}=R_{H_{SD},\;RX}^{1/2}H_{w_{SD}}R_{H_{SD},\;TX}^{1/2},\\ H_{{SR}_{l}}=R_{H_{{SR}_{l}},\;RX}^{1/2}H_{w_{{SR}_{l}}}R_{H_{{SR}_{l}},\;TX}^{1/2},\\ H_{R_{l}D}=R_{H_{R_{l}D},\;RX}^{1/2}H_{w_{R_{l}D}}R_{H_{R_{l}D},\;TX}^{1/2}. \end{array}\right.$$

where $H_{w_{SD}}$, $H_{w_{SR_{l}}}$, and $H_{w_{R_{l}D}}$ are full rank gain matrices of which the entries are i.i.d. and follow a circularly symmetric complex Gaussian distribution with zero-mean and unit variance. $R_{H_{SD},\;RX}$, $R_{H_{SR_{l}},\;RX}$ and $R_{H_{R_{l}D},\;RX}$ are the receiver correlation matrices. Finally, $R_{H_{SD},\;TX}$, $R_{H_{SR_{l}},\;TX}$ and $R_{H_{R_{l}D},\;TX}$ are the transmitter correlation matrices. Based on the exponential correlation model defined in [31,32], we model the entries of the receiver and transmitter correlation matrices presented in (13). In fact, for a correlation matrix, denoted R, the entries can be expressed as

(14) $${\left(R\right)}_{rc}=\left\{\begin{array}{c} \rho^{c-r},\;r\leq c\\ {\bar{\left(R\right)}}_{cr},\;r>c \end{array},\;\left|\rho \right|<1,\right.$$

where ρ denotes the amount of correlation.

### 2.3. Imperfect Channel Estimation Model

In practical cooperative MIMO systems, it is unfeasible to obtain perfect CSI at all nodes. In fact, errors can be produced by channel estimation, quantization, reciprocity mismatch, and delay. Consequently, it results in the presence of imperfect CSI. In this paper, we consider that the estimation of the backward $H_{{SR}_{l}}$ channels can be performed based on pilot signaling by $R_{l},l=1,\ldots ,L$ and thus relay nodes have a perfect knowledge of $H_{{SR}_{l}}$ [33]. However, we consider the existence of the imperfect CSI in SD and $R_{l}Dl=1,\ldots ,L$ links. Therefore, we model the imperfect CSI for SD and $R_{l}D,l=1,\ldots ,L$ links as [11]

(15) $$\left\{\begin{array}{c} {\hat{H}}_{SD}=H_{SD}+e_{SD}\Omega_{SD},\\ {\hat{H}}_{R_{l}D}=H_{R_{l}D}+e_{R_{l}D}\Omega_{R_{l}D}. \end{array}\right.$$

where the entries of $\Omega_{SD}$ and $\Omega_{R_{l}D}$ are i.i.d. zero-mean circularly symmetric complex Gaussian variables with unit variance. In addition, these matrices, i.e., $\Omega_{SD}$ and $\Omega_{R_{l}D}$, are independent of $H_{SD}$ and $H_{R_{l}D}$, respectively. $e_{SD}^{2}$ and $e_{R_{l}D}^{2}$ denote the estimation error variances of the SD and $R_{l}D$ channels, respectively. At the end of the second transmission phase, two copies of source data $x_{s}$ are received at *D* through the direct link SD, i.e., $y_{SD}$ Equation (6), and the cooperative links ${SR}_{l}-R_{l}D$, i.e., $y_{RD}$ (Equation (11)). These two copies are combined in order to increase the SNR. Therefore, the received signal at *D* without any time oversampling and optimum symbol timing and with perfect carrier frequency and phase estimation is given by

(16) $$\begin{array}{ccc} y_{D} & = & y_{SD}+y_{RD}. \end{array}$$

After that, discriminating features will be extracted from received signals as an input to the random committee MLT [34].

## 3. Modulation Detection Algorithm

### 3.1. Features Extraction

To correctly estimate the modulation from a received signal, an appropriate choice of key features is mandatory. The higher-order statistics (HOSs) that include the higher-order moments (HOMs) and higher-order cumulants (HOCs) are considered as promising features allowing to offer a good detection of modulation types [35,36]. For that reason, we choose the HOMs and HOCs statistics up to order eight for modulation detection purposes [36].

The *m*th-order HOM of a received signal vector at the *a*th antenna, denoted by $y_{D}^{\left(a\right)}=\left(\begin{array}{ccc} y_{D,1}^{\left(a\right)}, & \cdots & ,y_{D,N}^{\left(a\right)} \end{array}\right)$ can be written as [37]

(17) $$M_{mk}\left(y_{D}^{\left(a\right)}\right)=E\left[{\left(y_{D}^{\left(a\right)}\right)}^{m-k}{\left(\bar{y_{D}^{\left(a\right)}}\right)}^{k}\right],\;a=1,\ldots ,N_{AD}.$$

The HOMs can be also expressed as

(18) $${\hat{M}}_{mk}\left(y_{D}^{\left(a\right)}\right)=\frac{1}{N}\overset{N}{\sum_{n=1}}{\left(y_{D,n}^{\left(a\right)}\right)}^{m-k}{\left(\bar{y_{D,n}^{\left(a\right)}}\right)}^{k}.$$

The *m*th-order HOC of $y_{D}^{\left(a\right)}$ is given by

(19) $$C_{mk}\left(y_{D}^{\left(a\right)}\right)=Cum\left[\underset{\left(m-k\right)\;\mathrm{times}}{\underset{︸}{y_{D}^{\left(a\right)},\ldots ,y_{D}^{\left(a\right)}}},\underset{(k)\;\mathrm{times}}{\underset{︸}{y_{D}^{\left(a\right)},\ldots ,\;y_{D}^{\left(a\right)}}}\right].$$

where *m*th-order HOC can be written based on equal and lower ordered HOMs as [37]

(20) $$Cum\left[y_{D1}^{\left(a\right)},\ldots ,y_{Dm}^{\left(a\right)}\right]=\underset{\Psi }{\sum }{\left(-1\right)}^{\beta -1}\left(\beta -1\right)!\underset{\varphi \in \Psi }{\prod }E\left[\underset{c\in \varphi }{\prod }y_{Dc}^{\left(a\right)}\right],$$

where Ψ runs through the list of all partitions of 1,…,j, φ runs through the list of all blocks of the partition Ψ and β is the elements number of the partition Ψ. The interested reader can refer to the Appendix A for further details. We raise each HOC to the power 2/m as the magnitude of HOCs increases with their order [38].

To improve the performance of the proposed algorithm in terms of modulation detection and decrease the computational cost, a reduced set of features is chosen based on the principal component analysis (PCA) technique [39]. In fact, this latter technique allows building a low-dimensional representation of the extracted features that describes as much as possible the variance in that features. It represents a linear transformation that transforms the components of the extracted features to orthogonal components. Thereafter, it ranks the resulting orthogonal components in a manner that those with the largest variation are placed in the top of the list. Consequently, the selected subset of features is the orthogonal components with the largest variance, while the remaining components are those that present high correlations and thus can be removed with a minimal loss of information. Simulations show that only ten orthogonal components, i.e., $N_{feat}=10$, among twenty-eight, are chosen in the training and test phases.

**Figure 2:**
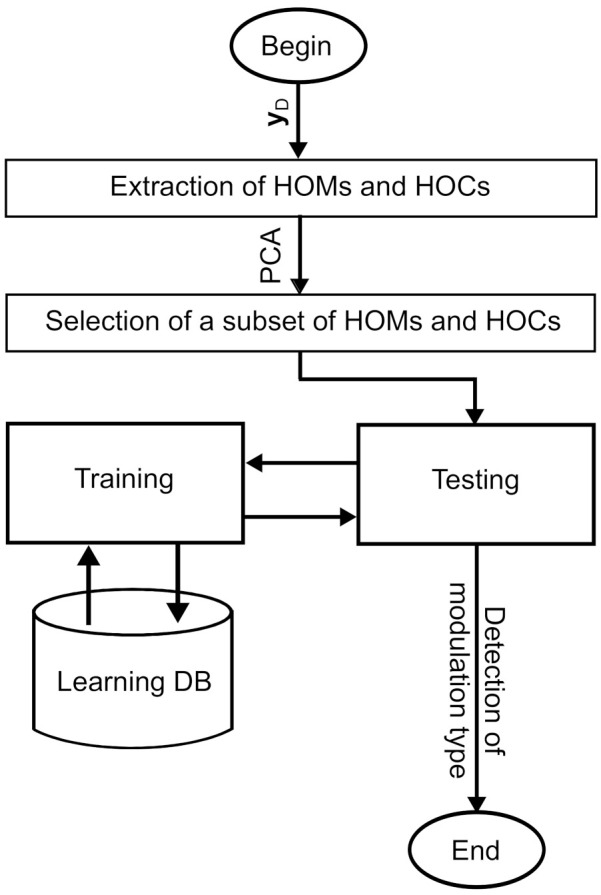
Modulation detection of a given $y_{D}$ signal.

To detect the modulation type of an unknown signal, denoted by $y_{D}$, a training phase should be launched. It involves building a classifier from a learning database (DB). Based on the built classifier, the test phase is done to detect the modulation type. In Figure 2, we present the modulation detection of a given $y_{D}$ signal.

### 3.2. Random Committee Operating with Random Tree MLT

To detect the modulation type used by the source node based on the received signals at the destination node, we deploy the random committee MLT [34] using the random tree as a base MLT [40]. Let *T* represents the number of the training subsets (i.e., $T_{1},\;\ldots ,\;T_{T}$). These latter construct an ensemble of $C_{1},\;\ldots ,\;C_{T}$ random tree classifiers, where each random tree classifier is formed based on a various random number seed using the same training data. With the use of the random tree classifier, a set of features is randomly selected in each node to construct the classifier and the final detection decision, denoted by $D_{final}$, is an average of the received predictions given by the individual random tree classifiers.

### 3.3. Adaptive Boosting (AdaBoost) Operating with Decision Tree

**Figure 3:**
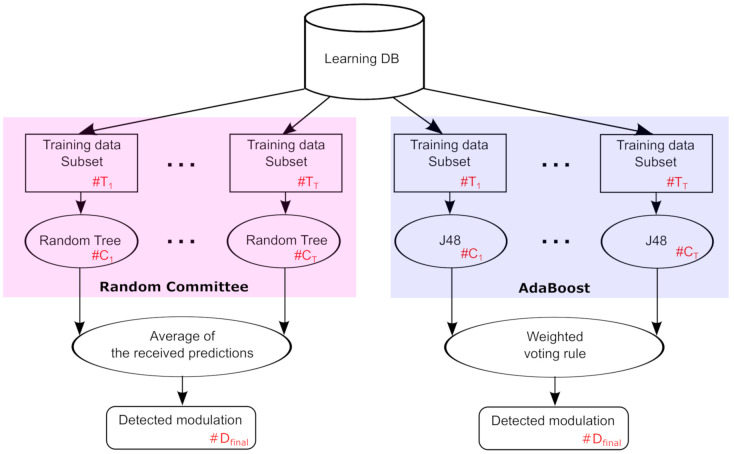
Random committee vs. AdaBoost processes.

In this work, we compare the random committee with the AdaBoost (Adaptive Boosting) MLT [41]. In fact, AdaBoost MLT produces a set of sequential decision tree (J48) classifiers. Thanks to classifiers that were previously built, it adjusts the weights of the training samples. Here, the goal is to force the J48 classifier to reduce expected errors under different input distributions. In fact, the training samples, that are wrongly detected by former classifiers, will play an essential role in the training of later ones. Using AdaBoost MLT, a number of *T* weighted training subsets $T_{1},\;\ldots ,\;T_{T}$ are created in sequence and *T* classifiers $C_{1},\;\ldots ,\;C_{T}$ are construct. Then, the final decision, denoted by $D_{final}$, is made based on the decision of $C_{1},\;\ldots ,\;C_{T}$ classifiers through a weighted voting rule. We notice that the weight of each classifier is set based on its performance on the training subset employed to construct it. In Figure 3, we present the random committee and AdaBoost processes.

Recall that *T* is the number of the generated training subsets, $N_{feat}$ is the number of selected features, and let $N_{samp}$ is the size of the learning DB, the time complexities of random committee using random tree MLT and AdaBoost using J48 MLT are given in Table 2.

### 3.4. Multilayer Perceptron (MLP)

We also compare the proposed algorithm using the random committee MLT with the MLP MLT used in [24]. In fact, MLP is one of the most widely used artificial neural networks (ANN). It uses the resilient backpropagation (RPROP) proposed in [42], which is known for its good performance on pattern recognition algorithms. Note that the structure of the MLP contains one input layer, one or more hidden layers and one output layer. Here, each neuron of a layer is linked to all the neurons of the next layer. The intuition behind the introduction of this hidden layer is to enable the network to model the functions of complex nonlinear decision between any input and output layers. The optimal MLP structure to be employed in this work is determined using intensive simulations. In fact, we show that MLP with two hidden layers excluding the input and the output layers, where the first layer contains 10 nodes and the second has 15 nodes, provides a good trade-off between modulation detection and training time. Consequently, we use MLP with these settings in our simulation.

### 3.5. Metrics Used for Performance Evaluation of MLTs

In this paper, we compare between Random committe and AdaBoost MLTs using true positive (TP) rate, false positive (FP) rate, precision, recall, and F-Measure metrics. The precision, recall, and F-measure are given, respectively, as

(21) $$\mathrm{precision}=\frac{\mathrm{TP}}{\mathrm{TP}+\mathrm{FP}},$$

(22) $$\mathrm{recall}=\frac{\mathrm{TP}}{\mathrm{TP}+\mathrm{FN}},$$

(23) $$F-\mathrm{measure}=2\times \frac{\mathrm{precision}\times \mathrm{recall}}{\mathrm{precision}+\mathrm{recall}}.$$

## 4. Simulation Results

The performance of the proposed algorithm was verified for multi-relay cooperative MIMO systems over spatially correlated channels through numerical simulations. The simulated modulations set contains the following list: $M=\left\{16QAM,\;64QAM,\;BPSK,\;QPSK\;\mathrm{and}\;8PSK\right\}$. A training set is built for each modulation type based on 50 realizations of signals with $512\times N_{A}$ symbols, where the messages transmitted by the source node and MIMO channels are randomly generated in each realization. We assume that all sub-channels, i.e., SD${SR}_{l}$, and $R_{l}D,l=1,\ldots ,L$, have the same correlation coefficient, i.e., $\left|\rho \right|=\left|\rho_{H_{ch},\;RX}\right|=\left|\rho_{H_{ch},\;TX}\right|,$ where $ch=\left(SD,\;{SR}_{l},\;R_{l}D\right)$. We also assume that the sub-channels SD and $R_{l}D$ have the same variance of estimation error, i.e., $e^{2}=e_{SD}^{2}=e_{R_{l}D}^{2}$. In all results, we consider that all nodes are equipped with four antennas, i.e., $N_{A}=4$. Added spatially white circularly complex Gaussian noises with variances $\sigma_{ch}^{2}$ are considered. Without loss of generality, we suppose that the sub-channels ${SR}_{l}$ have the same SNR, i.e., $SNR_{SR}=SNR_{{SR}_{l}}=20\;\mathrm{dB}$, l=1,2,…,L. Furthermore, we consider that the sub-channels SD and RD have equal SNRs, i.e., $SNR=SNR_{SD}=SNR_{RD}$.

In this work, we carry out a comparative study between the random committee MLT with the AdaBoost MLT using a 10-fold cross-validation [43] on the training set described above. Here, the number of training subsets is set to ten for both random committee and AdaBoost MLTs (i.e., T=10). For all the results, we consider a cooperative MIMO system with $N_{A}=4$, L=3, $SNR_{SR}=20\;\mathrm{dB}$, SNR=−5dB and $e^{2}=0.1$ where $\left|\rho \right|=0.5$. Here, we choose SNR=−5dB to evaluate the MLTs performance at low SNR as a good detection performance, i.e., 100%, can be achieved at acceptable SNR values as shown in our proposal presented in [21].

Table 3 and Table 4 display the detailed accuracy by modulation type for the random committee and the Adaboost MLTs, respectively, in the case of cooperative MIMO system with L=3, $N_{A}=4$, $SNR_{SR}=20\;\mathrm{dB}$, SNR=−5dB, $e^{2}=0.1$ and $\left|\rho \right|=0.5$. By comparing the average of the presented metrics, i.e., TP rate, FP rate, precision, recall, and F-Measure, it is clearly shown that the random committee MLT offers a gain compared to the Adaboost MLT in terms of modulation detection as the values of TP rate, precision, recall, and F-Measure of the random committee MLT are higher than the ones of the Adaboost MLT. However, the value of the FP rate for the random committee is lower than the one of the Adaboost MLT. Therefore, the random committee MLT can be adopted for modulation detection.

Table 5 and Table 6 confirm the obtained results. In fact, the percentages of correctly detected modulations are 84.86% and 84.285% for random committee and the Adaboost MLTs, respectively.

**Figure 4:**
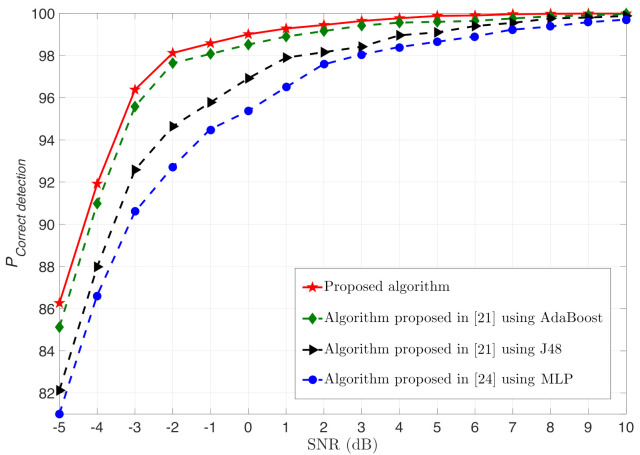
$P_{Correct\;detection}$ as a function of SNR for the proposed algorithm using random committee compared to MLP [24], algorithm proposed in [21] using J48 MLT alone and AdaBoost operating with J48, where L=3, $N_{A}=4$, $SNR_{SR}=20\;\mathrm{dB}$, $e^{2}=0.1$, and $\left|\rho \right|=0.5$.

To confirm these results, we show in Figure 4 the probability of correct detection ($P_{Correct\;detection}$) of the proposed algorithm using random committee MLT as a function of the SNR compared to the algorithm proposed in [21] using J48 MLT alone and AdaBoost MLT operating with J48, where L=3, $N_{A}=4$, $SNR_{SR}=20\;\mathrm{dB}$, $e^{2}=0.1$, and $\left|\rho \right|=0.5$. Here, we consider that the test set consists of 1000 Monte Carlo trials for each modulation scheme (i.e., 5000 Monte Carlo trials in total $N_{total}=5000$). For each trial, $N_{A}$ test signals are considered where each signal consists of 512 i.i.d. symbols. It clearly shown that the proposed algorithm provides a good performance, as $P_{Correct\;detection}$ reaches 100% (i.e., $P_{Correct\;detection}≃100\%$) at acceptable SNR. Furthermore, one can see that the modulation detection of our proposal is enhanced compared to the algorithm proposed in [21] for the two cases: using both the AdaBoost and J48 MLTs. For example, $P_{Correct\;detection}$ achieves about 100% at SNR equal to 5dB and 10dB for our proposal and the algorithm proposed in [21] using AdaBoost MLT, respectively. It is also shown that the MLP has the worst performance.

**Figure 5:**
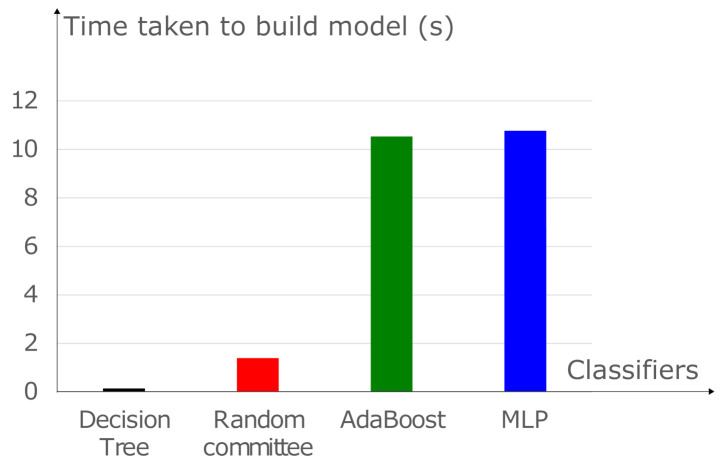
Time taken to build model: random committee Vs. AdaBoost Vs. J48 Vs. MLP with L=3, $N_{A}=4$, $SNR_{SR}=20\;\mathrm{dB}$, SNR=−5dB, $e^{2}=0.1$ and $\left|\rho \right|=0.5$.

In addition to the provided modulation detection gain, the random committee MLT has a low complexity compared to the Adaboost MLT. Indeed, the required time taken to build the model for the random committee is more than seven times faster than the Adaboost MLT as shown in Figure 5. One can also show that the training time of the MLP is long. Consequently, the proposed algorithm provides a good tradeoff between modulation detection performance and complexity.

## 5. Conclusions

In this paper, we studied the detection of modulations for the multi-relay cooperative MIMO systems in the presence of spatially correlated channels. At destination node, we extracted the higher-order statistics (HOSs) as discriminating features of the received signals. After applying the principal component analysis (PCA) technique, we carried out a comparative study between the random committee and the AdaBoost MLTs at low SNR. The efficiency metrics, including the true positive rate, false positive rate, precision, recall, F-Measure, and the time taken to build the model, are used for the performance comparison. Simulation results demonstrated that the use of the random committee MLT, as compared to the AdaBoost MLT, offers gain in terms of the complexity and modulation detection.

## Figures and Tables

**Table 1:** A list of abbreviations.

Abbreviation	Description
AdaBoost	Adaptive Boosting
ANN	Artificial neural networks
BM	Beamforming matrix
CSI	Channel state information
DB	Learning database
FP	False positive
HOCs	Higher order cumulants
HOMs	Higher order moments
HOSs	Higher order statistics
i.i.d.	Independent, identically distributed
LTE	Long term evolution
LTE-A	LTE-advanced
MLP	Multilayer perceptron
MLT	Machine learning technique
MIMO	Multiple-input multiple-output
RPROP	resilient backpropagation
RZF	Regularized zero forcing
SNR	Signal-to-noise ratio
SM	Spatial multiplexing
TP	True positive
ZF-RZF	Zero forcing and regularized zero forcing

**Table 2:** Time complexities of random committee using random tree classifier and AdaBoost using J48 MLT.

MLTs	Time Complexities
Random committe	$O\left(T\times N_{samp}\times log_{2}\left(N_{feat}\right)\right)$
AdaBoost	$O\left(T\times N_{samp}^{2}\times N_{feat}\right)$

**Table 3:** Detailed accuracy by modulation type for the random committee using random tree as a base MLT with L=3, $N_{A}=4$, $SNR_{SR}=20\;\mathrm{dB}$, SNR=−5dB, $e^{2}=0.1$, and $\left|\rho \right|=0.5$.

M	TP Rate	FP Rate	Precision	Recall	F-Measure
BPSK	0.952	0.014	0.945	0.952	0.949
QPSK	0.703	0.081	0.686	0.703	0.694
8PSK	0.652	0.071	0.696	0.652	0.674
16QAM	0.960	0.014	0.945	0.960	0.952
64QAM	0.976	0.010	0.962	0.976	0.969
**Avg.**	0.849	0.038	0.847	0.849	0.847

**Table 4:** Detailed accuracy by modulation type for Adaboost using J48 as a base MLT with L=3, $N_{A}=4$, $SNR_{SR}=20\;\mathrm{dB}$, SNR=−5dB, $e^{2}=0.1$, and $\left|\rho \right|=0.5$.

M	TP Rate	FP Rate	Precision	Recall	F-Measure
BPSK	0.951	0.013	0.949	0.951	0.950
QPSK	0.659	0.079	0.677	0.659	0.668
8PSK	0.663	0.083	0.667	0.663	0.665
16QAM	0.965	0.014	0.945	0.965	0.954
64QAM	0.978	0.008	0.967	0.978	0.972
**Avg.**	0.843	0.039	0.841	0.843	0.842

**Table 5:** Confusion matrix for random committee using random tree as a base MLT with L=3, $N_{A}=4$, $SNR_{SR}=20\;\mathrm{dB}$, SNR=−5dB, $e^{2}=0.1$, and $\left|\rho \right|=0.5$.

a	b	c	d	e	Detected as
3807	55	78	40	20	a = BPSK
75	2813	1026	54	32	b = QPSK
109	1211	2609	49	22	c = 8PSK
30	19	31	3838	82	d = 16QAM
6	5	2	82	3905	e = 64QAM
Correctly detected modulations				16,972	84.86%
Incorrectly detected modulations				3028	15.14%

**Table 6:** Confusion matrix for Adaboost using J48 as a base MLT with L=3, $N_{A}=4$, $SNR_{SR}=20\;\mathrm{dB}$, SNR=−5dB, $e^{2}=0.1$, and $\left|\rho \right|=0.5$.

a	b	c	d	e	Detected as
3803	62	82	39	14	a = BPSK
71	2635	1214	55	25	b = QPSK
107	1168	2650	56	19	c = 8PSK
21	24	21	3858	76	d = 16QAM
6	3	4	76	3911	e = 64QAM
Correctly detected modulations				16,857	84.285%
Incorrectly detected modulations				3143	15.715%

## Data Availability

The data are not publicly available due to project restrictions.

## Appendix A

Based on (17), the second-order HOM can be expressed as

(A1) $$M_{20}=E\left\{{\left(y_{D}^{\left(a\right)}\right)}^{2}\right\},\;\mathrm{and}\;M_{21}=E\left\{{\left|y_{D}^{\left(a\right)}\right|}^{2}\right\}.$$

$C_{20}$ and $C_{21}$ are written using (19) as

(A2) $$C_{20}\left(y_{D}^{\left(a\right)}\right)=\mathrm{Cum}\left[y_{D}^{\left(a\right)},y_{D}^{\left(a\right)}\right],\;C_{21}\left(y_{D}^{\left(a\right)}\right)=\mathrm{Cum}\left[y_{D}^{\left(a\right)},\bar{y_{D}^{\left(a\right)}}\right].$$

Assuming m=3, the available set of indices is $\left(1,\;2,\;3\right)$ and four distinct types of partitioning can be obtained for that set: $\left\{\left(1\right),\;\left(2\right),\;\left(3\right)\right\}$ leading to β=3, $\left\{\left(1\right),\;\left(2,\;3\right)\right\}$ leading to β=2, $\left\{\left(2\right),\;\left(1,\;3\right)\right\}$ leading to β=2, $\left\{\left(3\right),\;\left(1,\;2\right)\right\}$ leading to β=2, $\left\{\left(1,\;2,\;3\right)\right\}$ leading to β=1. Therefore,

(A3) $$\begin{array}{ccc} \mathrm{Cum}\left[y_{D1}^{\left(a\right)},y_{D1}^{\left(a\right)},y_{D3}^{\left(a\right)}\right] & = & {\left(-1\right)}^{1-1}\left(1-1\right)!E\left\{y_{D1}^{\left(a\right)}y_{D2}^{\left(a\right)}y_{D3}^{\left(a\right)}\right\}\;\;\;\;\;\;\;\\ \; & \; & +{\left(-1\right)}^{2-1}\left(2-1\right)!E\left\{y_{D1}^{\left(a\right)}\right\}E\left\{y_{D2}^{\left(a\right)}y_{D3}^{\left(a\right)}\right\}\;\;\;\;\;\\ \; & \; & +{\left(-1\right)}^{2-1}\left(2-1\right)!E\left\{y_{D2}^{\left(a\right)}\right\}E\left\{y_{D1}^{\left(a\right)}y_{D3}^{\left(a\right)}\right\}\;\;\;\;\;\\ \; & \; & +{\left(-1\right)}^{2-1}\left(2-1\right)!E\left\{y_{D3}^{\left(a\right)}\right\}E\left\{y_{D1}^{\left(a\right)}y_{D2}^{\left(a\right)}\right\}\;\;\;\;\;\;\\ \; & \; & +{\left(-1\right)}^{3-1}\left(3-1\right)!E\left\{y_{D1}^{\left(a\right)}\right\}E\left\{y_{D2}^{\left(a\right)}\right\}E\left\{y_{D3}^{\left(a\right)}\right\}\\ \; & = & E\left\{y_{D1}^{\left(a\right)}y_{D2}^{\left(a\right)}y_{D3}^{\left(a\right)}\right\}-E\left\{y_{D1}^{\left(a\right)}\right\}E\left\{y_{D2}^{\left(a\right)}y_{D3}^{\left(a\right)}\right\}\;\;\;\;\;\;\\ \; & \; & -E\left\{y_{D2}^{\left(a\right)}\right\}E\left\{y_{D1}^{\left(a\right)}y_{D3}^{\left(a\right)}\right\}-E\left\{y_{D3}^{\left(a\right)}\right\}E\left\{y_{D1}^{\left(a\right)}y_{D2}^{\left(a\right)}\right\}\\ \; & \; & +2E\left\{y_{D1}^{\left(a\right)}\right\}E\left\{y_{D2}^{\left(a\right)}\right\}E\left\{y_{D3}^{\left(a\right)}\right\}\;\;\;\;\;\;\;\;\;\;\;\;\;\;\;\;\;\;\;\;\; \end{array}$$

By following the same manner, one can express the HOC up to eighth order in terms of HOMs. For example, $C_{40}$, $C_{60}$, and $C_{80}$ are defined, respectively, as

(A4) $$C_{40}=M_{40}-3{\left(M_{20}\right)}^{2},$$

(A5) $$C_{60}=M_{60}-15M_{20}M_{40}+30{\left(M_{20}\right)}^{3},$$

(A6) $$C_{80}=M_{80}-35{\left(M_{40}\right)}^{2}-630{\left(M_{20}\right)}^{4}+420{\left(M_{20}\right)}^{2}M_{40}.$$
